# Eliminating Maternal and Neonatal Tetanus and Promoting Clean Delivery Practices Through Disposable Clean Birth Kits

**DOI:** 10.3389/fpubh.2019.00339

**Published:** 2019-11-21

**Authors:** Syed Ahsan Raza, Bilal Iqbal Avan

**Affiliations:** ^1^Department of Family and Community Medicine, Baylor College of Medicine, Houston, TX, United States; ^2^Faculty of Infectious and Tropical Diseases, London School of Hygiene and Tropical Medicine, London, United Kingdom

**Keywords:** neonatal tetanus, clean birth kits, neonatal infection, immunization (vaccination), infection

## Introduction

Tetanus is a vaccine-preventable acute disease manifested by instability of motor system and autonomic nervous system and is caused by a highly potent neurotoxin produced by the spore-forming bacterium *Clostridium tetani* that thrives in an anaerobic environment (1, 2). The spores of *C. tetani* are ubiquitous, found in soil and environment, and can contaminate wounds and abrasions. All mammals on land are affected by tetanus, and there is variation in susceptibility to the disease (2). Historically, it has been documented that primates such as monkeys, apes, and chimpanzees are more susceptible than carnivores (2, 3). In humans, the disease remains common in many low-resource countries where it represents a major prevention challenge. Although very rare in developed parts of world, it still presents a diagnostic and therapeutic challenge (2, 4).

Worldwide, tetanus contributes to a large proportion of maternal and neonatal deaths, estimated in 2008, to have caused approximately 180,000 deaths per year (1). In neonatal tetanus, the umbilical stump acts as an entry point for the bacteria after unhygienic delivery and cord care practices (2). In maternal tetanus, infection can occur after miscarriages, abortion, as well as unclean and hygienic delivery practices. Prevention is carried out through vaccination with tetanus toxoid, but since the spores of *C. tetani* are widespread in the environment, eradication is impossible. Therefore, the goal of global prevention strategies is to reach elimination of disease (2, 5).

## Maternal and Neonatal Tetanus Elimination

Neonatal tetanus (confirmed case) is defined as “a neonate with the normal ability to suck and cry during the first 2 days of life, and between 3 and 28 days of age cannot suck normally and becomes stiff or has spasms” (6). In the 1980s, even with the availability of cheap and effective prevention through maternal vaccination, high incidence rates were observed with high mortality (7). Hence, the World Health Assembly, in 1988, passed a resolution to eliminate neonatal tetanus by the year 2000, a disease that, at the time, was estimated to kill 800,000 neonates a year. The elimination of neonatal tetanus was realized as a grave public health problem and was defined as fewer than one case per 1,000 livebirths in every district or similar administrative unit in every country each year (8). By 1999, the World Health Organization (WHO), the United Nations Children's Fund (UNICEF), and the United Nations Population Fund (UNFPA) re-launched their efforts to achieve the goal of neonatal tetanus elimination. Since neonatal tetanus depends mainly on immunization of mother during pregnancy, the goal of elimination of maternal tetanus (defined as tetanus during pregnancy or within 6 weeks of the end of pregnancy) was also added to this initiative, which was then called “Maternal and Neonatal Tetanus Elimination Program.” The deadline for elimination was extended to 2005 and was set as the cutoff year to achieve this goal that later was again shifted to 2015 (8, 9). The fourth elimination goal for maternal and neonatal tetanus is now targeted for year 2020 (8).

Implementation of the Maternal and Neonatal Tetanus Elimination Initiative has involved two main strategies: *immunization* and *clean delivery and cord care practices* (and to much extent *surveillance* as well) (7, 8, 10). No formal reporting system exists for maternal tetanus, and elimination is assumed to be accomplished with elimination of neonatal tetanus (11–13). Mainly, due to high prevalence of inadequate immunization in some countries, along with unclean delivery services and inappropriate umbilical cord care, neonatal tetanus represents a high proportion of the total tetanus disease burden. Most of the neonatal deaths occur in countries from Sub-Saharan African regions and South Asia, generally in areas where poverty is rampant. In some of these regions, the main problem for mothers is access to quality antenatal health care, in addition to inadequate information about clean delivery practices, since most of the deliveries happen in home (14). Traditional practices in home deliveries that includes application of harmful substances to umbilical stump still persist even in pockets of poor localities of large urban cities where access to health care facilities are supposedly better (15–17). The fatality rate of neonatal tetanus can be as high as 100% in case of home deliveries or where deliveries are not carried out in proper health care facilities (12, 16). In many developing countries, the extent of burden cannot be estimated since many neonates and women die during these home deliveries and where there is no appropriate system of surveillance through which both the birth and death can be reported (5).

Neonatal tetanus is historically neglected as a health problem in developing countries (9). This is not only because it is a disease of poor that has been eliminated by many countries of the world, but for the reason that it can still pose a serious public health challenge in countries where the marginalized and displaced population sometimes lack a strong political representation, e.g., Afghan refugees in Pakistan and some of the federally administered tribal areas bordering Pakistan and Afghanistan (18–21). Traditional attitudes of people in the past such as considering deaths from neonatal tetanus as wish of God and viewing immunization campaigns with suspicion have likely contributed to the neglect in some regions of the world (9, 22, 23). Of late, natural disasters, armed conflicts, and politically motivated fake vaccination programs by vested interests are other important factors that have disrupted a properly functioning public health infrastructure and have caused additional challenges for global prevention and elimination strategies of childhood vaccine preventable diseases including neonatal tetanus (2, 24–27).

**Figure 1:**
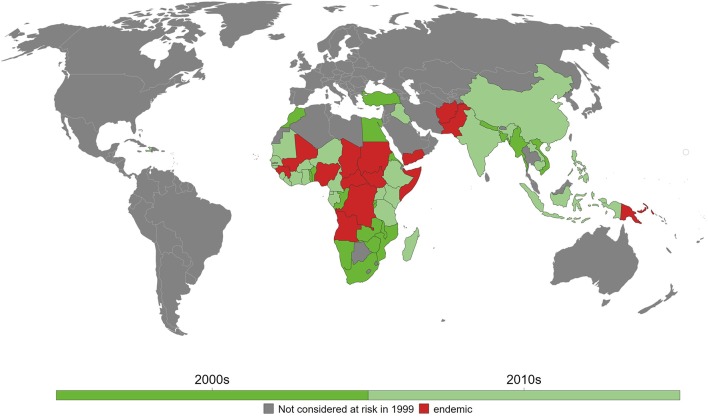
Maternal and neonatal tetanus (MNT) elimination in the last two decades. As of 2018, 14 countries have yet to eliminate MNT. Reprinted from Behrens et al. (28) with permission.

To date, 14 countries have yet to achieve elimination goal in 2020 and where maternal and neonatal tetanus remains a big challenge mainly due to wars, conflicts, and politically vulnerable environment. These include Afghanistan, Angola, Central African Republic, Chad, Congo, Guinea, Mali, Nigeria, Pakistan, Papua New Guinea, Somalia, Sudan, South Sudan, and Yemen (Figure 1) (28). India, as one of the largest South Asian countries, achieved the elimination goal in 2015 (29), whereas, in Africa, Kenya became one of the last countries in 2018 that has been declared free of maternal and neonatal tetanus (8).

## Immunization

**Figure 2:**
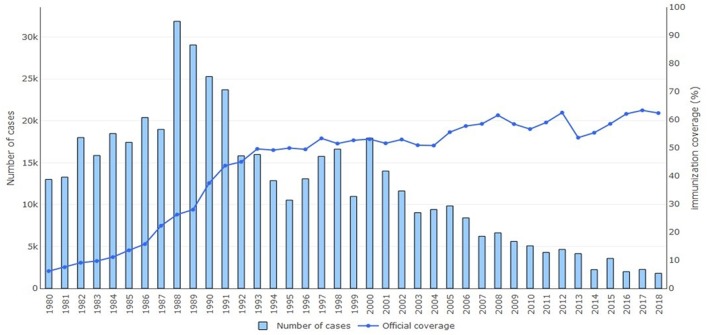
Annual reported cases of neonatal tetanus and coverage of two or more doses of tetanus toxoid vaccine, 1980–2018. Reprinted with permission from World Health Organization (6).

One of the two main strategies for eliminating neonatal tetanus and, as a consequence, maternal tetanus is *immunization*. Tetanus toxoid vaccine is available as a monovalent tetanus toxoid, a bivalent toxoid combined with diphtheria or reduced diphtheria toxoid content, or a trivalent vaccine combined with diphtheria and whole cell or acellular pertussis (DTP vaccine). Other vaccine combinations with hepatitis B, *Hemophilus influenzae* type b, and polio also exists (2, 30). As part of the Expanded Program on Immunization, WHO has recommended three doses of DTP vaccine to an infant at 2, 3, and 4 months followed by boosters at 4–7 years and 15 years of age (2, 31). To prevent neonatal tetanus, maternal immunization is recommended with two doses of tetanus toxoid 4 weeks apart during pregnancy for women who have never been vaccinated or incompletely vaccinated. In order for long-lasting maternal protection, a total of five doses should be given; a third dose should be given 6 months after the second dose and then two subsequent doses should be given 5 and 10 years later. Maternal vaccination provides protection for an estimated 84% of the neonates (8, 13). Figure 2 shows the percentage of maternal immunization coverage with two doses of tetanus toxoid vaccine from 1980 to 2018 in countries where neonatal tetanus is still a major public health problem. Since 2000, a decline is seen in reported cases of neonatal tetanus with this immunization coverage even though surveillance of cases may still be incomplete in resource-limited countries.

The elimination initiative for maternal and neonatal tetanus uses two immunization approaches. The first approach most commonly used is the *routine immunization* of pregnant women that aims to deliver two doses of tetanus toxoid 1 month apart. The second approach, known as *supplementary immunization activities* (*SIAs*) are employed in areas deemed to be high risk for neonatal tetanus where the first approach may not be effective. In SIAs, opportunities for vaccination are provided beyond the conventional antenatal settings such as school-based programs, markets, and community-based settings. Worldwide, the additional application of SIAs has been helpful in making significant progress in many countries between 1999 and 2013 (7, 32, 33). Since maternal and neonatal tetanus occurs more in communities where there is high prevalence of unclean home deliveries practiced by traditional birth attendants, the two immunization approaches are likely to have greatest impact in the low-resource regions of the world. While supplementary immunization have been successful in increasing the coverage of vaccines in low-resource regions, particularly in conflict zones such as in Afghanistan and Somalia, these activities have recently been found to be unintentionally detrimental and disruptive to the routine health services (34). The authors have called for greater support for routine services to complement supplementary health services.

## Clean Birth Kits

The second elimination strategy for maternal and neonatal tetanus is improving birth hygiene or practicing clean deliveries. The importance of clean practices during delivery has been emphasized for centuries from times of Ancient Greeks and from texts of Avicenna (35). One of the best examples in this regard is the case of child-bed fever or “puerperal sepsis” realized by nineteenth-century Hungarian physician Ignaz Semmelweis, when he demonstrated that an approach as simple as “cleaning hands” could prevent totally unnecessary deaths of mothers during delivery. The epidemic nature of infectious causes of maternal and neonatal deaths and its prevention through clean delivery practices was realized however much later after Semmelweis's death in 1865 (35, 36). Many decades later, in 1998, WHO published a review of evidence for care of umbilical cord and summarized clean delivery and cord care practices as “six cleans” (37).

High neonatal mortality rates are observed in developing countries due to neonatal umbilical cord infections. Preventions of these infections involve practicing “six cleans” along with increased coverage of tetanus toxoid immunization. However, in regions of the world where vaccination and immunization are now viewed with suspicion (24, 26), promotion of these six clean practices is a pragmatic step. A few elementary supplies are required for carrying out these practices, e.g., soap to wash hands and perineum, a clean blade to cut the umbilical cord, and clean thread to tie the cord (14, 38). Scarcity of these six clean supplies, however, may be a hindering factor in some settings, owing to “supply chain bottlenecks” or because women cannot afford to pay. However, on many occasions, when these supplies are available, there is a need for complex behavioral change to make sure that delivery attendants or traditional birth attendants in the community practice these six cleans and that there is cultural acceptability to women and their families. Therefore, universal access to clean and hygienic child delivery requires addressing the bottlenecks on both the supply and demand side of the health system. Some interventions exert effects through multiple channels, for instance, with clean birth kits, which may influence both content of care and uptake (14, 17, 38).

**Figure 3:**
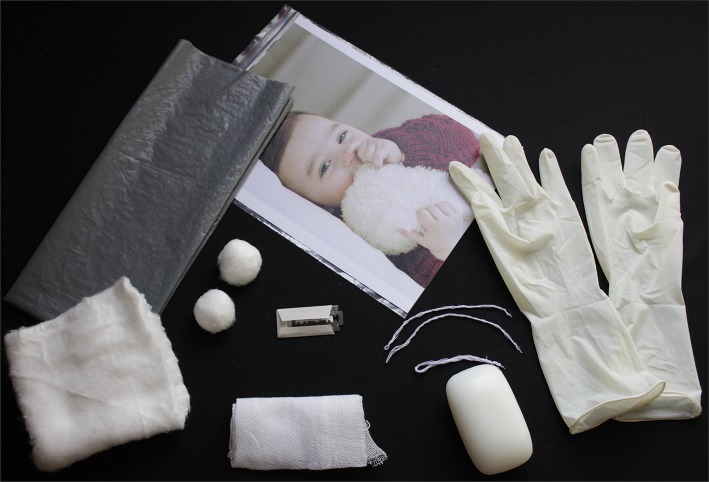
Contents of Clean Delivery Kit to ensure clean delivery practices that includes plastic delivery sheet, a soap, blade, cord ties/ligand, spirited gauze, and, additionally, gloves (credits: Bushra Khan and Waqas Hameed).

WHO has been endorsing supply of clean birth kits that are disposable in resource-poor settings for many decades (39, 40). This is seen as a cost-effective way of providing minimum necessary birthing provisions to promote hygienic deliveries and cord care (14). These kits consist of very basic supplies for performing clean deliveries that are helpful, especially in remote areas where a hospital delivery cannot be carried out (17, 41–45). Clean birth kits are also highly encouraged in hospital facilities where sometimes hygienic practices are not always possible because of improper sterilization techniques and limited supply of water (17). The main utility of these disposable kits is to provide minimum but necessary supplies for carrying out a clean delivery in one affordable package, which can be cheaply available to mothers and birth attendants. The contents of these sterilized disposable packages facilitate six clean practices, which are (1) plastic delivery sheet for clean surface, (2) a soap to ensure clean hands and (3) perineum, (4) a blade for clean cutting of umbilical cord, (5) cord ties/ligand for clean tying of cord, and, finally, (6) gauze and spirit for clean post-delivery cord care. Most of these kits also consist of a pair of gloves for carrying out clean delivery. The contents of these kits can be seen in Figure 3.

Availability of skilled birth attendants such as doctors, nurses, and trained midwives who have access to clean services in health care facilities are crucial in reducing neonatal deaths. While availability of skilled attendants is accepted as benchmark in many countries, this alone is not enough to bring down neonatal mortality in a short period of time in less developed countries (17). Again, to reinforce the point, regions where there is chronic lack of skilled attendants, and where traditional birth attendants practice deliveries, and where there is not enough access to clean delivery services and where water supply is in short supply, clean birth kit seems a cost-effective strategy. Meta-analyses and literature reviews on use of disposable kits (14, 38, 42, 46) have indeed provided clues on an increase in clean practices by attendants, even when it is as simple as cleaning of hands during and after child delivery. Lower incidence rates of range of infectious outcomes have been observed such as in cases of omphalitis and postpartum infections (47). By conventional standards as well as historically, the use of these cost-effective kits by birth attendants in remote community settings have shown to reduce the incidence of some of the neonatal and maternal infections in China and India (10, 48).

## Discussion

Maternal and neonatal tetanus cannot be eradicated because of the ubiquitous nature of the bacterium in the environment, and therefore continuous efforts are needed to invest in vaccination programs for its elimination. However, in some lower- to middle-income countries of the world, these elimination programs through vaccination are likely to face multiple serious challenges on a similar pattern as in the case of other vaccine-preventable diseases (22, 23, 49). In Pakistan and Afghanistan, for example, maternal and neonatal tetanus will remain an unfinished agenda as long as there are conflict issues at the border areas along with challenges in other areas of these countries, such as inadequate surveillance, false and superstitious beliefs, high level of distrust with health care personnel, and inaccessibility to areas with militancy. Hence, new and innovative interventions are particularly needed in these low-resource settings.

In many countries, maternal and neonatal tetanus were eliminated through clean delivery practices even before vaccines were introduced; e.g., clean cord care practices through skilled delivery assistance have been shown to be an effective intervention in reducing neonatal mortality by 50–70% (50). In China, the success of eliminating maternal and neonatal tetanus in 2012 was credited to clean delivery practices and increased facility-based deliveries, without specific vaccination schemes (51). A study carried out among the Maasai tribe in East Africa has shown that interventions to improve delivery practices sharply reduced mortality from maternal and neonatal tetanus, even without vaccination programs (52). While clean practices have clearly shown to be effective in reducing mortality in regions where most of the deliveries are carried out in homes, maintaining these practices even with skilled attendants remains challenging.

Clean birth kits to ensure clean practices have shown to be a promising strategy in reducing maternal and neonatal morbidity and mortality (38, 42, 53, 54). A systematic review by Blencowe et al. suggests that these kits to improve clean practices are highly cost-effective, at an estimated US$ 215 per life saved (14). Our previous study from Pakistan reports that provision of skilled attendants and clean birth kits is independently associated with reduction in neonatal tetanus. The population attributable risk for not using birth kit was 24%, which means that approximately one-quarter of neonatal cases can be prevented with the use of these kits (17). In regions of the world with poor coverage of tetanus toxoid immunization, clean birth kits appear to be an effective strategy to achieve Sustainable Development Goals of reducing maternal and neonatal mortality.

The main limitation in literature regarding clean delivery practices through clean birth kits has been the lack of good quality data assessing the effect of these kits on neonatal outcomes. Since it would not be ethical to randomize expectant mothers to receive or not receive birth kits during the time of delivery, there are very few randomized trials highlighting the effectiveness of these kits. A clustered randomized trial carried out in rural Pakistan examined the effects of training traditional birth attendants with the supply of clean birth kits; however, the specific contribution of kit usage in reducing mortality could not be estimated (55). Evidence is however available regarding the use of innovative interventions such as chlorhexidine wash or wipes (for umbilical cord cleansing) compared to acceptable standard practices (15). One major challenge is that it is difficult to separate the effect of birth kits or practices from other interventions in some of these reports such as tetanus toxoid vaccinations and other health promotion approaches (14). Certain contextual/confounding factors can also affect the generalizability of these results of published reports.

Based on pooled estimates from epidemiological studies and extensive review by Blencowe et al. (38), facility-based birth compared to home birth was found to reduce the risk of death from neonatal tetanus by 70%, after controlling for major confounders such as immunization coverage. Blencowe's meta-analysis also found that intervention studies that included clean birth kits were associated with improved outcomes in neonatal tetanus and omphalitis. The evidence for the effect of these kits on neonatal outcomes was however not high and the results may not be easily generalizable. All studies in the analyses included clean birth kits as part of evaluation package alongside other interventions using different delivery mechanisms and with different background characteristics, e.g., current practices, and background tetanus rates, etc.

Even though clean birth kits have shown to have the potential to improve quality of care at birth, some questions still remain. One important issue to understand is whether mothers using the kits act as a disincentive for giving birth in the hospitals. Further information can be gained through new surveys and analyses of existing research datasets in developing countries. Another important public health priority is regarding the question of cost and health system impact of adding more contents in birth kits such as disinfectants like chlorhexidine or labor-inducing medications such as misoprostol for highly trained delivery attendants. Here, “Implementation Science Research” will be needed for these add-ons for additional contents. For these add-ons, extreme caution should be practiced; e.g., labor-inducing medications should not be available to untrained delivery attendants. Then, there is also a question of facility-based kits in low-resource settings that could be much more extensive with the inclusion of a blood pressure device and a suction device with other essential drugs. Implementation research will again be required to examine advantages in their efficiency over alternative approaches.

Because of the increasing provision of vaccines, under-five mortality has been declining. However, in order to further accelerate progress toward Sustainable Development Goals in developing regions of the world, a major focus must be placed on increasing *coverage of immunization* and quality of antenatal care. This, however, requires a very proactive approach to invest in *human resources* of developing countries such as health care workers who have deep roots with their communities and who are well equipped with essential supplies for basic, safe, and clean delivery practices.

Clean birth kits have already been made available in many developing countries; however, as pointed out in Blencowe's policy brief (5), robust evaluations are lacking regarding their contribution. Moreover, these kits are also life savers in *conflict or humanitarian emergencies* or in settings where facility-based clean deliveries are not always possible. For countries that are already planning to promote clean birth kits or adding new contents, it will be crucial to collect data on their experiences, e.g., implementation techniques, impact, as well as costs involved.

## Author Contributions

SR carried out the literature review and wrote the first draft of the manuscript. BA provided critical revisions of the manuscript.

### Conflict of Interest

The authors declare that the research was conducted in the absence of any commercial or financial relationships that could be construed as a potential conflict of interest.
